# The Critical Role of AMPKα1 in Regulating Autophagy and Mitochondrial Respiration in IL-15-Stimulated mTORC1^Weak^ Signal-Induced T Cell Memory: An Interplay between Yin (AMPKα1) and Yang (mTORC1) Energy Sensors in T Cell Differentiation

**DOI:** 10.3390/ijms23179534

**Published:** 2022-08-23

**Authors:** Anjuman Ara, Zhaojia Wu, Aizhang Xu, Khawaja Ashfaque Ahmed, Scot C. Leary, Md. Fahmid Islam, Rajni Chibbar, Yue Wu, Jim Xiang

**Affiliations:** 1 Cancer Research Cluster, Saskatchewan Cancer Agency, 20 Campus Drive, Saskatoon, SK S7N 4H4, Canada; 2 Division of Oncology, College of Medicine, University of Saskatchewan, 107 Wiggins Road, Saskatoon, SK S7N 5E5, Canada; 3 Department of Pathology, Western College of Veterinary Medicine, University of Saskatchewan, Saskatoon, SK S7N 5B4, Canada; 4 Department of Biochemistry, Microbiology and Immunology, College of Medicine, University of Saskatchewan, 107 Wiggins Road, Saskatoon, SK S7N 5E5, Canada; 5 Department of Pathology, College of Medicine, University of Saskatchewan, 107 Wiggins Road, Saskatoon, SK S7N 5E5, Canada

**Keywords:** IL-15, mTORC1, AMPKα1, FOXO1, T-cell memory, autophagy, mitochondrial biogenesis, fatty acid oxidation

## Abstract

Two common γ-chain family cytokines IL-2 and IL-15 stimulate the same mammalian target of rapamycin complex-1 (mTORC1) signaling yet induce effector T (T_E_) and memory T (T_M_) cell differentiation via a poorly understood mechanism(s). Here, we prepared in vitro IL-2-stimulated T_E_ (IL-2/T_E_) and IL-15-stimulated T_M_ (IL-15/T_M_) cells for characterization by flow cytometry, Western blotting, confocal microscopy and Seahorse-assay analyses. We demonstrate that IL-2 and IL-15 stimulate strong and weak mTORC1 signals, respectively, which lead to the formation of CD62 ligand (CD62L)^−^ killer cell lectin-like receptor subfamily G member-1 (KLRG)^+^ IL-2/T_E_ and CD62L^+^KLRG^−^ IL-15/T_M_ cells with short- and long-term survival following their adoptive transfer into mice. The IL-15/mTORC1^Weak^ signal activates the forkhead box-O-1 (FOXO1), T cell factor-1 (TCF1) and Eomes transcriptional network and the metabolic adenosine monophosphate-activated protein kinase-α-1 (AMPKα1), Unc-51-like autophagy-activating kinase-1 (ULK1) and autophagy-related gene-7 (ATG7) axis, increasing the expression of mitochondrial regulators aquaporin-9 (AQP9), mitochondrial transcription factor-A (TFAM), peroxisome proliferator-activated receptor-γ coactivator-1α (PGC1α), carnitine palmitoyl transferase-1 (CPT1α), microtubule-associated protein light chain-3 II (LC3II), Complex I and ortic atrophy-1 (OPA1), leading to promoting mitochondrial biogenesis and fatty-acid oxidation (FAO). Interestingly, AMPKα1 deficiency abrogates these downstream responses to IL-15/mTORC1^Weak^ signaling, leading to the upregulation of mTORC1 and hypoxia-inducible factor-1α (HIF-1α), a metabolic switch from FAO to glycolysis and reduced cell survival. Taken together, our data demonstrate that IL-15/mTORC1^Weak^ signaling controls T-cell memory via activation of the transcriptional FOXO1-TCF1-Eomes and metabolic AMPKα1-ULK1-ATG7 pathways, a finding that may greatly impact the development of efficient vaccines and immunotherapies for the treatment of cancer and infectious diseases.

## 1. Introduction

CD8^+^ T cells play an important role in immunity against infection [1]. In early infection, the stimulation of naïve CD8^+^ T cells induces their proliferation and differentiation into two T cell subsets with distinct expression of the memory T (T_M_) cell marker IL-7 receptor (IL-7R) and the senescent effector T (T_E_) cell marker killer cell lectin-like receptor subfamily G member-1 (KLRG1) [2]. The IL-7R^−^KLRG1^+^ short-lived effector cells (SLECs) then terminally differentiate into T_E_ cells poised for cell apoptosis during the contraction phase, while the IL-7R^+^KLRG1^−^ memory precursor effector cells (MPECs) survive the contraction phase and differentiate into quiescent CD8^+^ T_M_ cells [2]. MPECs have extended survival potential that is supported by elevated FAO and exhibit robust recall responses upon pathogen recounter, properties which protect the host from secondary infection [1]. This T_M_-cell population is comprised of three cell subsets that have been phenotypically well characterized: CD45RA^+^IL-7R^+^CD62L^+^ stem cell-like memory T (T_SCM_) cells derived from MPECs, and IL-7R^+^CD62L^+^ central T_M_ (T_CM_) and IL-7R^+^CD62L^−^ effector T_M_ (T_EM_) cells, both of which are derived from CD45RA^+^IL-7R^+^CD62L^+^ T_SCM_ cells [2].

Various transcription factors and kinases crucial to controlling T-cell phenotype and differentiation have been identified. The transcription factors forkhead box-O-1 (FOXO1), T-cell factor-1 (TCF1), inhibitor of DNA binding-3 (Id3), cMyb and Eomes all play important roles in T_M_-cell differentiation [3,4]. The adenosine monophosphate-activated protein kinase-α1 (AMPKα1), an evolutionarily conserved energy sensor, is crucial for controlling cellular metabolism and survival by activating various regulators of autophagy and mitochondrial respiration to meet increased energetic demands. Autophagy is a self-recycling process that occurs in the cytosol in which proteins and organelles are degraded via lysosomes to provide essential precursors for anabolism [5]. AMPKα1 promotes flux through autophagy via the activation of Unc-51-like autophagy-activating kinase-1 (ULK1), autophagy-related gene-7 (ATG7) and microtubule-associated protein light chain-3 II (LC3II; an autophagosome maturation marker). AMPKα1 also promotes the fusion of individual mitochondria by inducing a shift from dynamin-related protein-1 (DRP1)-regulated fission events to optic atrophy-1 (OPA1)-controlled mitochondrial fusion events, which in turn stimulates the expression of several players critical to mitochondrial biogenesis and a switch in fuel preference to fatty acid oxidation (FAO). These AMPKα1 targets include peroxisome proliferator-activated receptor-γ coactivator-1α (PGC1α), aquaporin-9 (AQP9), carnitine palmitoyl transferase-1α (CPT1α), Complex I of electron transport chain (ETC) and mitochondrial transcription factor-A (TFAM) [4,5,6]. The mammalian target of rapamycin complex-1 (mTORC1) is another evolutionarily conserved energy sensor that controls the activity of ribosomal S6 kinase (S6K) and eukaryotic translation initiation factor-4E (eIF4E), two downstream substrates crucial to the modulation of T-cell proliferation, differentiation, metabolism and survival [7]. mTORC1 also activates the transcription factors Blimp1, Id2 and T-bet, which are pivotal to the T_E_-cell phenotype and differentiation, as well as cMyc and hypoxia-inducible factor-1 (HIF-1α), which are central to the reliance of T_E_ cells on the glycolytic metabolism [3,4,7]. However, AMPKα1- and mTORC1-dependent molecular pathways that regulate CD8^+^ T-cell memory have yet to be fully explored.

The common γ-chain (γ_c_) cytokines, such as the pro-inflammatory IL-2 and the pro-survival IL-7 and IL-15, are crucial for T-cell expansion, differentiation and survival [8]. The potent and selective in vivo stimulation of CD8^+^ T-cell memory by IL-15 was first shown by Sprent and colleagues in 1998 [9]. Subsequent reports provided additional evidence in support of their original finding, by demonstrating that IL-15 quantitatively and qualitatively promotes the formation and maintenance of a CD8^+^ T_M_-cell pool [10,11,12]. IL-15 was further shown to promote T-cell memory via the activation of multiple downstream targets for T-cell differentiation that include the transcriptional factor TCF1 [13,14], the mitochondrial fusion factor OPA1 [15], lysosomal acid lipase (LAL) [16] and CPT1α, an enzyme critical to FAO [17]. However, the molecular pathway(s) underlying IL-15-mediated T-cell memory has yet to be elucidated.

It has been shown that the pro-inflammatory IL-2 and pro-survival IL-7 exert distinct effects on T-cell differentiation, even though they trigger the same JAK3 (Janus kinase-3)-activated PI3K (phosphatidylinositol-3 kinase)-AKT-mTORC1 signaling pathway. However, the underlying molecular mechanism by which IL-2 stimulates the formation of short-lived T_E_ cells while IL-7 promotes the formation of long-lived T_M_ cells [8,18] remains elusive. Recently, we provided the first evidence that the pro-inflammatory cytokine IL-2 and the pro-survival cytokine IL-7 distinctly stimulate distinct strengths of mTORC1 (IL-2/mTORC1^Strong^ and IL-7/mTORC1^Weak^) signaling. We showed that IL-2/mTORC1^Strong^ signal stimulation in IL-2/T_E_ cells results in persistent expression of the surface receptor IL-2Rα, whereas IL-7Rα is transiently expressed at the cell surface as a result of IL-7/mTORC1^Weak^ signal stimulation in IL-7/T_M_ cells. Thus, while both IL-2 and IL-7 trigger the same JAK3-activated PI3K-AKT-mTORC1 signaling pathway, it is the unique mTORC1 signal strengths that ultimately lead to the formation of short-lived T_E_ and long-lived T_M_ cells [18]. In addition, we demonstrate that the IL-7 stimulated mTORC1^Weak^ signal promotes the formation of T_M_ cells via the coupled activation of the transcriptional FOXO1-TCF1-Id3 and metabolic AMPKα1-ULK1-ATG7 pathways, which respectively regulate the T_M_-cell phenotype and FAO metabolism [18]. To assess whether this novel finding is specific to IL-7 or represents a general means by which pro-survival cytokines induce T-cell memory, similar studies of the well-characterized cytokine IL-15 are warranted.

This study therefore aims to elucidate the molecular pathways controlling IL-15-induced T-cell memory. To address this issue, we systematically characterize in vitro prepared IL-2- and IL-15-cultivated T (IL-2/T_E_ and IL-15/T_M_) cells derived from ovalbumin (OVA)-specific T-cell receptor (TCR) transgenic OTI mice using the methodology known to approximate the differentiation of T_E_ and T_M_ cells in vivo [16,18,19,20]. We demonstrated that IL-2/T_E_ and IL-15/T_M_ cells display IL-2/mTORC1^Strong^ and IL-15/mTORC1^Weak^ signaling and become short-term IL-7R^−^CD62L^−^KLRG1^+^ T_E_ cells and long-lived IL-7R^+^CD62L^+^KLRG1^−^ T_M_ cells, respectively, upon adoptive transfer into B6.1 mice. To identify the molecular pathways that support the formation of IL-15/T_M_ cells, we performed a suite of molecular and biochemical analyses using IL-2/T_E_ and IL-15/T_M_ cells and showed that IL-15/mTORC1^Weak^ signaling in IL-15/T_M_ cells activates the transcriptional FOXO1-TCF1-Eomes pathway regulating T_M_ cell differentiation, and the metabolic AMPKα1-ULK1-ATG7 pathway controlling autophagy, mitochondrial biogenesis and FAO metabolism. To assess the critical role of the energy sensor AMPKα1 in IL-15-induced T cell memory, we genetically engineered *AMPKα1* knock-out (KO)/OTI mice and prepared in vitro IL-15-cultivated T_M_ cells expressing or lacking AMPKα1 (AMPKα1 KO). Using these cells, we demonstrated that AMPKα1 deficiency abolishes the expression of autophagic ULK1, but up-regulates mTORC1 signaling and stimulates the mTORC1-controlled transcription factor HIF-1α, leading to a metabolic switch from FAO to glycolysis in AMPKα1 KO IL-15/T_M_ cells. Finally, we show that AMPKα1 KO IL-15/T_M_ cells lose their long-term survival following adoptive transfer into B6.1 mice and exhibit impaired recall responses upon antigen challenge.

## 2. Results

### 2.1. IL-2 and IL-15 Stimulate the Differentiation of CD8^+^CD62L^−^KLRG1^+^ T_E_ and CD8^+^CD62L^+^KLRG1^−^ T_M_ Cells, Which Have Short- and Long-Term Survival Potential after Adoptive Transfer into C57BL/6 Mice

**Figure 1:**
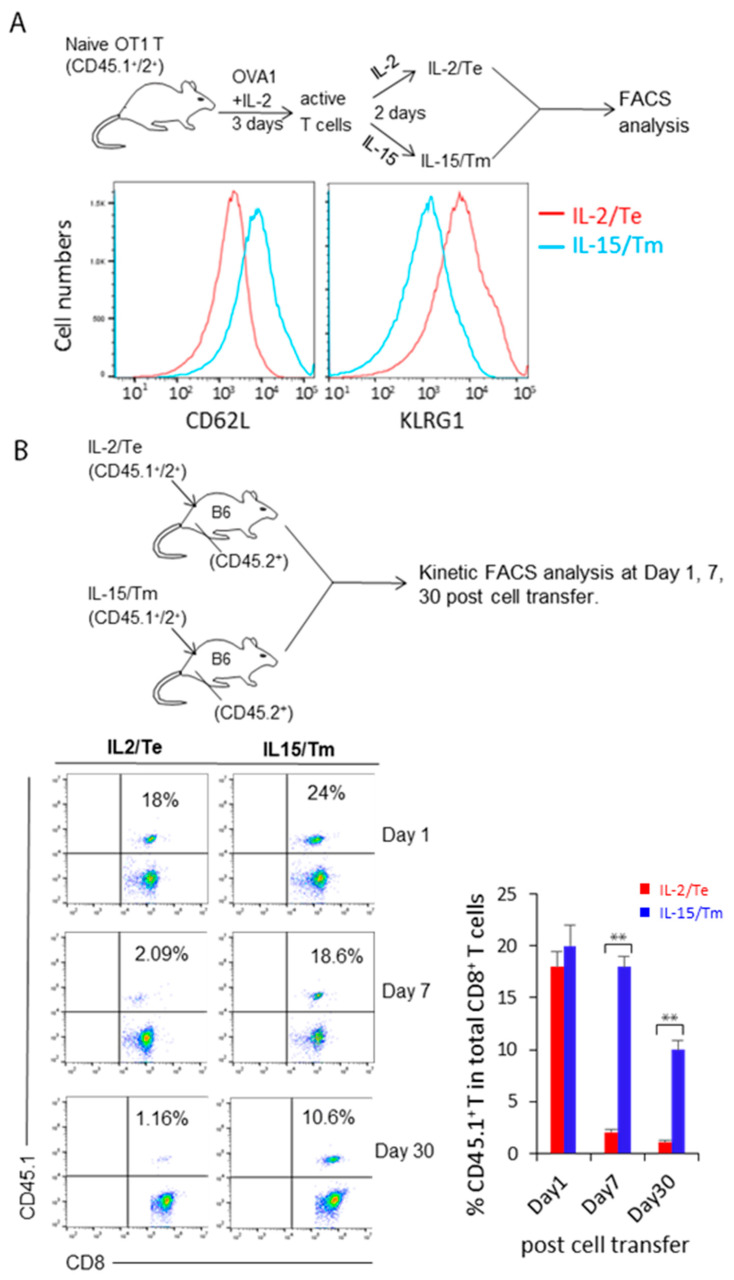
Characterization of in vitro prepared IL-2/T_E_ and IL-15/T_M_ cells by flow cytometry. (**A**) Schematic diagram of our experimental protocol for flow cytometry analysis of CD62L and KLRG1 expression. Naïve B6.1 (CD45.1^+^/2^+^)/OTI CD8^+^ T cells were cultured in complete media containing IL-2 and OVA1 peptide for 3 days, and subsequently transferred to complete media supplemented with IL-2 or IL-15 for an additional 2 days to form IL-2/T_E_ and IL-15/T_M_ cells. T cells were then stained with anti-CD62L and KLRG1 antibodies for flow cytometry analysis. (**B**) Schematic diagram of our experimental protocol for kinetic flow cytometry analysis of T-cell survival post-adoptive transfer. IL-2/T_E_ or IL-15/T_M_ cells were adoptively and separately transferred into B6 (CD45.2^+^) mice, and their relative abundance was quantified by flow cytometry at days 1, 7 and 30 post T cell transfer. Bar graphs show the donor CD8^+^ T cells expressed as a percentage of total host CD8^+^ T cells. Data are presented as mean ± SD (*n* = 3/group). ** *p* < 0.01 by two-tailed Student *t* test. A representative experiment of two is shown.

We first used a well-established protocol to prepare active T_E_ and T_M_ cells by stimulating CD8^+^ T cells from OVA-specific TCR transgenic OTI mice in vitro with IL-2 and IL-15, which approximates the in vivo T_E_- and T_M_-cell differentiation programs and allowed us and others to generate a large amount of T cells amenable to systematic characterization [16,18,19,20]. Briefly, we generated IL-2/T_E_ and IL-15/T_M_ cells by in vitro culture of naïve CD8^+^ T cells derived from CD45.1^+^/45.2^+^ wild-type (WT) OTI mice with OVA1 (SIINFEKL) peptide and IL-2 for 3 days. Activated T cells were then cultured with IL-2 or IL-15 for 2 more days to form IL-2/T_E_ and IL-15/T_M_ cells, respectively, and the expression of the memory T-cell marker CD62L and the effector T-cell marker KLRG1 was then measured by flow cytometry [18]. These analyses revealed that IL-15 stimulated cells harbor a T_M_ cell phenotype (CD62L^high^ and KLRG1^low^) (Figure 1A). The reciprocal expression pattern for these cell surface markers was observed in IL-2/T_E_ cells (Figure 1A). To determine whether IL-15/T_M_ cells exhibit better survival in vivo, we adoptively, but separately transferred an equal amount of in vitro-prepared IL-2/T_E_ or IL-15/T_M_ cells derived from CD45.1^+^/CD45.2^+^ WT OTI mice into CD45.1^+^ B6.1 mice. We then performed flow cytometry to kinetically analyze T-cell survival, as previously described [18], and found significantly more donor IL-15/T_M_ cells than IL-2/T_E_ cells in B6.1 mouse peripheral blood at days 7 and 30 post T-cell transfer (Figure 1B). These results indeed suggest that IL-15/T_M_ cells survive longer in host mice than IL-2/T_E_ cells.

### 2.2. IL-2 and IL-15 Binding Induce Sustained IL-2Rα and Transitional IL-15Rα Expression, Leading to Distinct mTORC1 Signaling Strengths in IL-2/T_E_ and IL-15/T_M_ Cells

**Figure 2:**
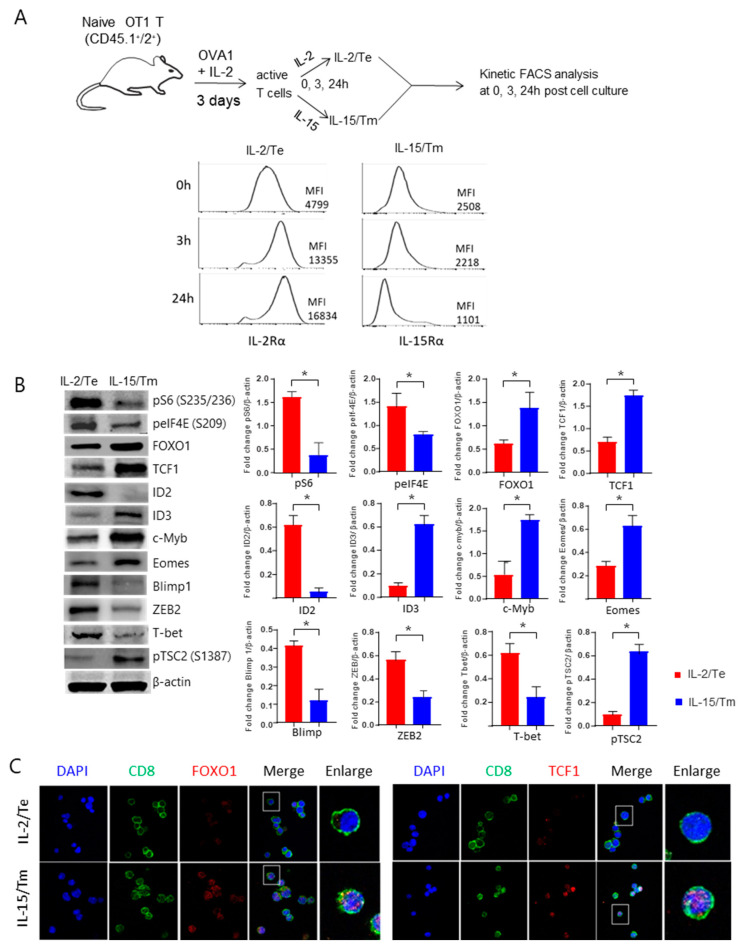
Characterization of in vitro prepared IL-2/T_E_ and IL-15/T_M_ cells by flow cytometry, Western blotting and confocal microscopy analyses. (**A**) Schematic diagram of the experimental protocol for in vitro prepared IL-2/T_E_ and IL-15/T_M_ cells derived from WT CD45.1^+^/2^+^ B6.1/OTI mice for kinetic flow cytometry measurement of cell surface expression of IL-2Rα and IL-15Rα at 0 h, 3 h and 24 h in culture. MFI represents the mean fluorescence intensity. (**B**) Cell lysates derived from IL-2/T_E_ and IL-15/T_M_ cells were analyzed by Western blotting to quantify the abundance of proteins of interest. Bar graphs represent the fold change in a given protein normalized to β-actin levels. Data are presented as mean ± SD (*n* = 3/group). * *p* < 0.05 by two-tailed Student *t* test. (**C**) Immunofluorescence analysis of the subcellular localization of FOXO1 and TCF1 (red) in IL-2/T_E_ and IL-15/T_M_ cells. DAPI (blue) was used as a counterstain for nuclei. Magnification, 100×. One representative experiment of the two is depicted.

It was demonstrated that after binding to their receptors, the cytokines IL-2 and IL-15 confer strong and weak stimuli to T cells due to sustained IL-2Rα and transitional IL-15Rα expression, respectively [12,21]. To test whether our in vitro prepared IL-2/T_E_ and IL-15/T_M_ cells behave similarly, we measured the cell surface expression of IL-2Rα and IL-15Rα over time in activated T cells cultured in media supplemented with IL-2 or IL-15. Indeed, we found that IL-2 stimulation enhanced IL-2Rα expression in IL-2/T_E_ cells, whereas IL-15 stimulation down-regulated IL-15Rα expression in IL-15/T_M_ cells (Figure 2A). To assess the effect of IL-2 and IL-15-stimulation on the strength of mTORC1 signaling, we performed Western blot analysis of lysates derived from in vitro prepared IL-2/T_E_ and IL-15/T_M_ cells (Figure 1A) to quantify the relative abundance of two mTORC1 substrates; phosphorylated ribosomal S6 kinase (pS6; S_235/236_) and phosphorylated eIF4E (peIF4E; S_209_). The abundance pS6 and peIF4E was upregulated in IL-2/T_E_ cells, but very low in IL-15/T_M_ cells (Figure 2B), indicating that IL-2/T_E_ and IL-15/T_M_ cells display mTORC1^Strong^ and mTORC1^Weak^ signaling, respectively.

### 2.3. IL-15-Stimulated CD8^+^ T_M_ Cells with mTORC1^Weak^ Signaling Activate the Transcriptional FOXO1-TCF1-Eomes Pathway

To further characterize the downstream responses to IL-15 stimulation of mTORC1^Weak^ signaling, we quantified the relative abundance of several transcription factors with known roles in T_E_ and T_M_ cell formation by Western blotting. IL-15/T_M_ cells with mTORC1^Weak^ signaling had significantly higher levels of FOXO1, TCF1, Eomes, cMyb and Id3 crucial for T_M_-cell phenotype and differentiation, while the abundance of Blimp1, Id2, T-bet, ZEB2 and cMyc central to T_E_-cell phenotype, and differentiation [3,4] was significantly lower (Figure 2B). IL-2/T_E_ cells with mTORC1^Strong^ signaling exhibited the reciprocal transcription factor expression profile (Figure 2B). In addition, IL-2/mTORC1^Strong^ signaling led to the accumulation of transcriptional factor ZEB2 that coordinates T-bet’s effect on T_E_-cell differentiation [22] in IL-2/T_E_ cells, while the mTORC1 suppressor pTSC2 (S_1387_) accumulated in IL-15/T_M_ cells as a result of IL-15/mTORC1^Weak^ signaling (Figure 2B). It was demonstrated that FOXO1 is an upstream regulator controlling TCF1 and Eomes [3,18]. Therefore, our data indicate IL-2/mTORC1^Strong^ and IL-15/mTORC1^Weak^ signaling stimulates the differentiation of IL-2/T_E_ and IL-15/T_M_ cells via activation of the transcriptional FOXO1-TCF1-Eomes and T-bet pathways, respectively. Non-phosphorylated FOXO1 represents the active form of the protein localized to the nucleus, while pFOXO1 re-localizes to the cytoplasm, where it is subsequently degraded upon poly-ubiquitination [23]. The FOXO1 substrate TCF1 also plays a controlling role in T_M_-cell formation through its nuclear localization [18]. Therefore, to visualize their subcellular localization at a single-cell level, we conducted confocal microscopy analyses using anti-FOXO1 and anti-TCF1 antibodies [18] and found that both proteins localized to the nucleus to a greater extent in IL-15/T_M_ than IL-2/T_E_ cells (Figure 2C).

### 2.4. mTORC1^Weak^ Signaling in IL-15-Stimulated CD8^+^ T_M_ Cells Activates the Metabolic AMPKα1-ULK1-ATG7 Pathway

**Figure 3:**
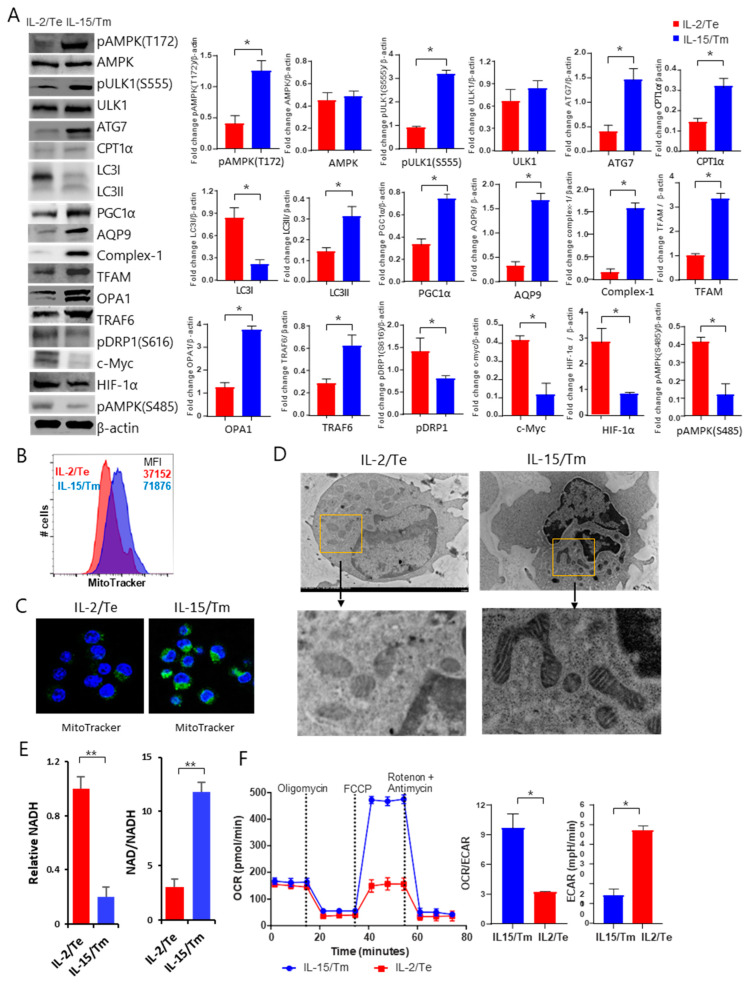
Characterization of in vitro prepared IL-2/T_E_ and IL-15/T_M_ cells by Western blotting, flow cytometry, confocal and electron microscopy and Seahorse-assay analyses. (**A**) Cell lysates derived from IL-2/T_E_ and IL-15/T_M_ cells were analyzed by Western blotting to quantify the abundance of proteins of interest. Bar graphs represent the fold change in a given protein normalized to β-actin levels. Data are presented as means ± SD (*n* = 3/group). * *p* < 0.05 by two-tailed Student *t* test. (**B**) IL-2/T_E_ and IL-15/T_M_ cells stained with Mitotracker were analyzed by flow cytometry to measure mitochondrial content. MFI represents the mean fluorescence intensity. (**C**) IL-2/T_E_ and IL-15/T_M_ cells were stained with Mitotracker and imaged by confocal microscopy to visualize mitochondria (green). Nuclei are Hoechst stained (blue). Magnification, 100×. (**D**) IL-2/T_E_ and IL-15/T_M_ cell pellets were fixed, sectioned, stained and checked under electron microscope, and mitochondrial morphology was visualized by electron microscopy analysis. Magnification, 1200×. (**E**) The relative amount of NADH and the NAD/NADH ratio in IL-2/T_E_ and IL-15/T_M_ cells. (**F**) Seahorse assay analyses of ECAR, OCR and the OCR/ECAR ratio under basal conditions and in response to applying various mitochondrial inhibitors in IL-2/T_E_ and IL-15/T_M_ cells. The above data represent one of two independent experiments and are presented as mean ± SD (*n* = 3/group). * *p* < 0.05, ** *p* < 0.01 by two-tailed Student *t* test.

Autophagy represents a self-recycling process whereby cellular constituents are degraded within lysosomes to provide essential anabolic precursors for cells to maintain energy homeostasis, particularly under stress conditions [24]. ULK1 and ATG7 are two major components of the autophagy pathway [25]. Phosphorylation of the energy sensor AMPKα1 (pAMPKα1) at T_172_ leads to the phosphorylation of ULK1 (pULK1) at S_555_ and the activation of ATG7, which in turn promotes autophagic flux [26]. In addition, LC3II is a commonly used marker for autophagy activity [26]. We therefore assessed the expression of pAMPKα1 (T_172_) and other regulators of T cell metabolism by Western blot analysis. We found that IL-15/mTORC1^Weak^, but not IL-2/mTORC1^Strong^, signaling indeed stimulated an increase in the levels of pAMPKα1 (T_172_), pULK1 (S_555_) and ATG7 in IL-15/T_M_ cells, compared to similar levels of AMPKα1 and ULK1 expression in IL-2/T_E_ and IL-15/T_M_ cells (Figure 3A). We also demonstrated that IL-15/T_M_ cells elevated the level LC3-II as well as several mitochondrial proteins crucial for FAO, as shown in the section below, while down-regulating the abundance of the master regulator of glycolysis HIF-1α [27] (Figure 3A). The reciprocal expression profile of these proteins was observed in IL-2/T_E_ cells (Figure 3A). These data indicate that IL-15/mTORC1^Weak^ signaling promotes T-cell memory in IL-15/T_M_ cells via activation of the metabolic AMPKα1-ULK1-ATG7 pathway, while IL-2/mTORC1^Strong^ signaling induces T_E_-cell formation in IL-2/T_E_ cells via activation of the metabolic HIF-1α pathway.

### 2.5. mTORC1^Weak^ Signaling in IL-15-Stimulated CD8^+^ T_M_ Cells Enhances Mitochondrial Biogenesis

We next elected to characterize several mitochondrial proteins that were shown to support mitochondrial biogenesis in T cells by Western blot analysis. These proteins include TFAM for mitochondrial respiration [16], AQP9 for the import of glycerol for fatty acid esterification and triacylglycerol (TAG) synthesis essential for mitochondrial programming and biogenesis [28], PGC1α, a critical regulator for mitochondrial biogenesis [29], CPT1α, a key regulator for FAO [17], and tumor necrosis factor (TNF) receptor-associated factor-6 (TRAF6) which modulates FAO via activation of AMPK1α [30]. Cristae are invaginations of the inner mitochondrial membrane that are enriched for the complexes of OXPHOS vital to mitochondrial respiratory competence [31]. The mitochondrial-shaping proteins are a family of proteins that control mitochondrial morphology and dynamics. Among them, a group of GTP-dependent dynamin-like proteins regulate the opposing processes of organelle fusion and fission, which are important to FAO and glycolysis, respectively, DRP1 [31]. To further investigate the importance of OXPHOS, mitochondrial fusion and fission to the T_M_-cell phenotype, we selected a marker of each biochemical pathway. We chose OPA1 because it is a GTPase that controls the fusion of the inner mitochondrial membrane and configures ETC complex associations to promote oxidative phosphorylation (OXPHOS) [15]. We selected Complex I (type I NADH dehydrogenase) based on the fact it is the largest of the dually encoded, multi-subunit enzymes critical to pumping protons from the mitochondrial matrix into the intermembrane space to create an electrochemical proton gradient necessary for the generation of ATP [32]. Finally, we also characterized DRP1, given its essentiality to mitochondrial fission [32].

To assess whether activation of the metabolic AMPK-ULK1-ATG7 pathway affects the expression of these mitochondrial proteins, we conducted Western blot analysis using IL-2/T_E_ and IL-15/T_M_ cell lysates. Indeed, we demonstrated that IL-15/T_M_ cells harbored more TFAM, AQP9, PGC1α CPT1α, Complex I and OPA1 for mitochondrial biogenesis and fusion but less pDRP1 (S_616_), which is essential for mitochondrial fission [15] (Figure 3A). The reciprocal protein abundance profile was observed in IL-2/T_E_ cells (Figure 3A), indicating that IL-15/ mTORC1^Weak^ signaling induces T-cell memory by promoting mitochondrial biogenesis. In addition, IL-2/mTORC1^Strong^ signaling enriched for pAMPK1α (S_485_) in IL-2/T_E_ cells, while IL-15/mTORC1^Weak^ signaling promotes the accumulation of the AMPK1α activator TRAF6 in IL-15/T_M_ cells [30]. To confirm the stimulatory effect of IL-15/mTORC1^Weak^ signaling on mitochondrial content, we conducted flow cytometry and confocal microscopy analyses using MitoTracker Green, a dye that binds specifically to mitochondrial membranes [18,19]. Indeed, we found that IL-15/T_M_ cells had higher mitochondrial content than IL-2/T_E_ cells (Figure 3B,3C). Recently, changes in mitochondrial morphology via the remodeling of fission and fusion events were shown to be associated with a preference for glycolysis in the small, round mitochondria of T_E_ cells and FAO in the tubular organelles of T_M_ cells [28]. Consistent with the idea that mitochondrial morphology heavily influences fuel preference in T cells, our electron microscopy analysis revealed that IL-15/T_M_ and IL-2/T_E_ cells harbored elongated/tubular and small/round mitochondria, respectively (Figure 3D).

### 2.6. IL-15-Stimulated CD8^+^ T_M_ Cells with an mTORC1^Weak^ Signal Have Substantial Mitochondrial SRC and Rely on FAO

Reactive oxygen species (ROS) and NADH generated by OXPHOS and the tricarboxylic acid (TCA) cycle impinge upon mitochondrial respiration and influence T-cell metabolism and proliferation [33,34]. In this study, we therefore used NAD+/NADH assay kits to measure NADH levels as a marker of glycolysis and the ratio of NAD+/NADH as a readout of FAO in IL-15/T_M_ cells.

Consistent with the idea that IL-15/T_M_ cells rely on FAO, we observed a significant enrichment in the abundance of Complex I in IL-15/T_M_ cells compared to IL-2/T_E_-cells (Figure 3A). We then quantified the total amount of NAD^+^ and NADH. IL-15/T_M_ cells had a lower amount of NADH, but a higher NAD/NADH ratio than IL-2/T_E_ cells (Figure 3E), suggesting that IL-15/T_M_ cells have greater OXPHOS flux capacity. Mitochondria are bioenergetic organelles that contribute to energy homeostasis and T_M_-cell survival [35] via OXPHOS and spare respiratory capacity (SRC), which are both essential for FAO [19,36]. To directly assess energy metabolism, we examined the bioenergetic profiles under basal conditions and after blocking flux through ETC Complexes I (rotenone) or III (antimycin A) or inhibiting (oligomycin) or uncoupling (FCCP) ATP synthesis in IL-2/T_E_ and IL-15/T_M_ cells. We found that IL-15/T_M_ cells produced more ATP (Figure 3F). IL-15/T_M_ cells also had a higher ratio of OCR (O_2_ consumption rate)/ECAR (extracellular acidification rate) than IL-2/T_E_ cells (Figure 3F), indicating that IL-15/T_M_ cells prefer FAO for energy production. In contrast, IL-2/T_E_ cells favor glycolysis as evidenced by an increased basal ECAR, a marker of glycolysis, and a lower level of OCR compared to IL-15/T_M_ cells (Figure 3F). Taken together, our data indicate that IL-15/T_M_ cells have substantial SRC to produce ATP via OXPHOS and utilize FAO to preserve energy homeostasis.

### 2.7. AMPKα1 Deficiency in IL-15-Stimulated CD8^+^ T_M_ Cells Impairs Mitochondrial Biogenesis and Induces a Metabolic Switch from FAO to Glycolysis

**Figure 4:**
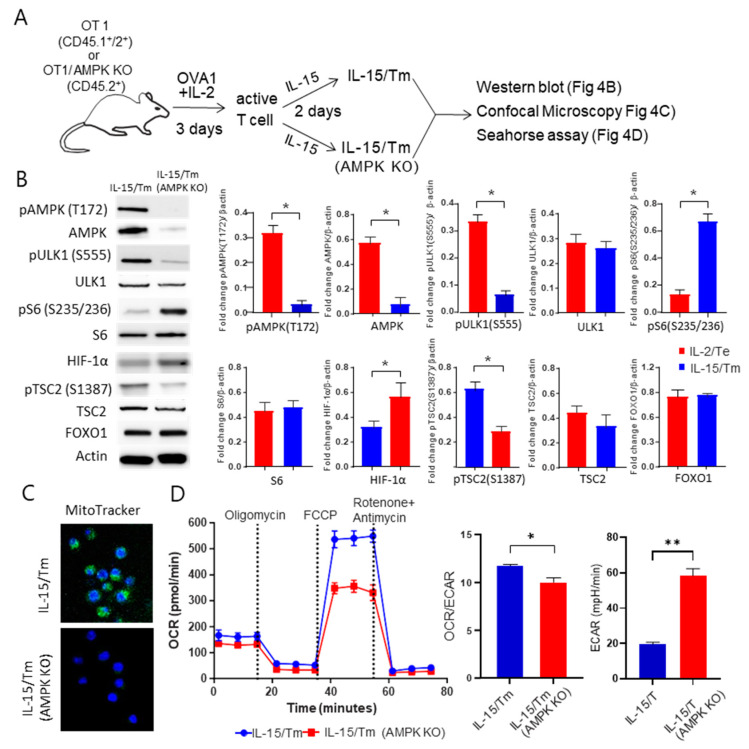
Characterization of in vitro prepared WT IL-15/T_M_ and IL-15/T_M_ AMPKα1 KO cells. (**A**) Schematic diagram of the experimental design for in vitro culture of WT and AMPKα1 KO IL-15/T_M_ analyzed by Western blotting, confocal microscopy and Seahorse assays. (**B**) The abundance of proteins of interest in WT and AMPKα1 KO IL-15/T_M_ cells. Bar graphs represent the fold change in a given protein normalized to β-actin levels. (**C**) WT and AMPKα1 KO IL-15/T_M_ cells were stained with Mitotracker Mitotracker and imaged by confocal microscopy to visualize mitochondria (green). Magnification, 100×. The nuclei are Hoechst stained (blue). (**D**) Seahorse assay analysis was performed to measure ECAR, OCR and the OCR/ECAR ratio under basal conditions or in response to applying various mitochondrial inhibitors in WT and AMPKα1 KO IL-15/T_M_ cells, respectively. All data representing one of two independent experiments are presented as means ± SD (*n* = 3/group). * *p* < 0.05, ** *p* < 0.01 by two-tailed Student *t* test.

To confirm the critical regulatory role of AMPKα1 in reliance of IL-15/T_M_ cells on FAO, we repeated the above experiments using in vitro prepared WT IL-15/T_M_ and AMPKα1 KO IL-15/T_M_ cells derived from CD45.1^+^/45.2^+^ WT OTI and CD45.2^+^ *AMPKα1* KO/OTI mice, respectively (Figure 4A). We then performed Western blot analysis to assess whether AMPKα1 deficiency affects the abundance of key autophagic and metabolic markers. We found that while the deletion of AMPKα1 in IL-15/T_M_ cells did not affect FOXO1 abundance, it did reduce the expression of both pAMPKα1 (T_172_) and AMPK as well as the autophagic pULK1 (S_555_) and pTSC2 (S_1387_), but did not affect the expression of ULK1, S6 and TSC2 (Figure 4B), consistent with our previous report [18]. AMPKα1 KO IL-15/T_M_ cells also had less mitochondrial mass (Figure 4C), and lower rates of FAO using the OCR/ECAR ratio as a proxy (Figure 4D). Interestingly, AMPKα1 deficiency up-regulated the abundance of the mTORC1 substrate pS6 (S_235/236_) kinase and the mTORC1-regulated transcription factor HIF-1α essential for glycolysis (Figure 4B). Consistent with these observations, AMPKα1 KO IL-15/T_M_ cells had increased ECAR, which is indicative of enhanced glycolysis (Figure 4E). These data indicate that loss of AMPKα1 induces a metabolic switch from FAO to glycolysis in AMPKα1 KO IL-15/T_M_ cells.

### 2.8. AMPKα1 Deficiency Impairs IL-15/T_M_ Cell Survival and Recall Responses

**Figure 5:**
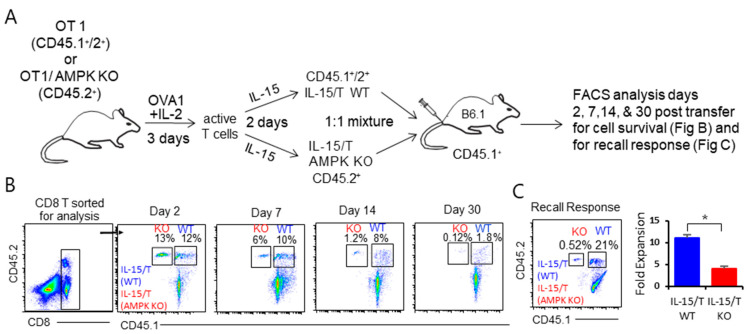
Characterization of the survival and recall responses of in vitro prepared WT IL-15/T_M_ and IL-15/T_M_ AMPKα1 KO cells. (**A**) Schematic diagram depicting our experimental protocol of adoptive T cell transfer and downstream, kinetic flow cytometric analyses. (**B**) A 1:1 mixture of in vitro prepared WT CD45.1^+^/2^+^ IL-15/T_M_ and CD45.2^+^ IL-15/T_M_ AMPKα1 KO cells derived from WT CD45.1^+^/2^+^ OTI and CD45.2^+^ OTI/*AMPKα1* KO mice was adoptively transferred into one CD45.1^+^ B6.1 mouse. Kinetic flow cytometry was performed by first sorting the host CD8^+^ T cell population for further analysis of the two donor CD45.1^+^/2^+^ WT IL-15/T_M_ and CD45.2^+^ AMPKα1 KO IL-15/T_M_ cells at day 2, 7, 14 and 30 post T cell transfer. (**C**) Recipient mice were infected with 2000 CFUs of rLmOVA at day 30 post T cell transfer. Representative dot plots showing frequencies of transferred CD45.1^+^/45.2^+^ WT IL-15/T_M_ and CD45.2^+^ AMPKα1 KO IL-15/T_M_ cells among the host CD8^+^ T cells by flow cytometry at day 4 after rLmOVA challenge. Bar graphs represent fold expansion of the T cell numbers at day 4 post the rLmOVA boost versus the T-cell numbers at day 30 post T-cell transfer. Data are presented as mean ± SD (*n* = 3/group). * *p* < 0.05 by two-tailed Student *t* test. One representative experiment of two is shown.

To assess whether AMPKα1 deficiency affects IL-15/T_M_-cell survival, a 1:1 mixture of WT CD45.1^+^/45.2^+^ IL-15/T_M_ and AMPKα1 KO CD45.2^+^ IL-15/T_M_ cells were adoptively transferred into the same CD45.1^+^ B6.1 mouse and followed kinetically by flow cytometry to measure transferred T-cell survival (Figure 5A). The advantage of this approach is that it allows us to distinguish between WT and AMPKα1 KO IL-15/T_M_ cells in the same host mouse, and therefore simultaneously track their survival. Using this method, we found a comparable amount of WT (12%) and AMPKα1 KO (13%) IL-15/T_M_ cells in mouse peripheral blood 2 days post T-cell transfer. However, 14 and 30 days after adoptive transfer, we observed significantly fewer AMPKα1 KO IL-15/T_M_ cells (1.2% and 0.12%) than WT IL-15/T_M_ cells (8% and 1.8%), with WT IL-15/T_M_ being 15-fold enriched over AMPKα1 KO IL-15/T_M_ at day 30 post T-cell transfer (Figure 5B). To measure their recall responses, the host mice were i.v. challenged with rLmOVA at day 30 post cell transfer, followed by flow cytometry analysis 4 days later. These analyses showed that the ability of AMPKα1 KO IL-15/T_M_ cells to expand during the recall response was impaired relative to WT IL-15/T_M_ cells (4.3- vs. 11.5-fold).

## 3. Discussion

CD8^+^ memory T (T_M_) cells play a critical role in immune defense against infection. Stimulation of naïve CD8^+^ T cells with three major immunomodulatory signals (antigen, co-stimulation and cytokine) triggers the PI3K-AKT-mTORC1 pathway and induces T-cell proliferation and differentiation into T_E_- and T_M_-cell subsets post infection. A fundamental question in immunity is the origin of the long-lived T_M_ cells. A linear cell differentiation (LCD) model was originally proposed by Sallusto’s group in 2000, in which different weak and strong strengths of immune stimuli regulate T-cell differentiation into long-term T_M_ and short-lived T_E_ cells, respectively [37]. Accumulating evidence strongly supports this well-known model of different strengths of stimuli in programming T_E_- and T_M_-cell differentiation [4]. However, the underlying molecular mechanism(s) regulating the distinct T-cell differentiation programs in LCD remains elusive.

**Figure 6:**
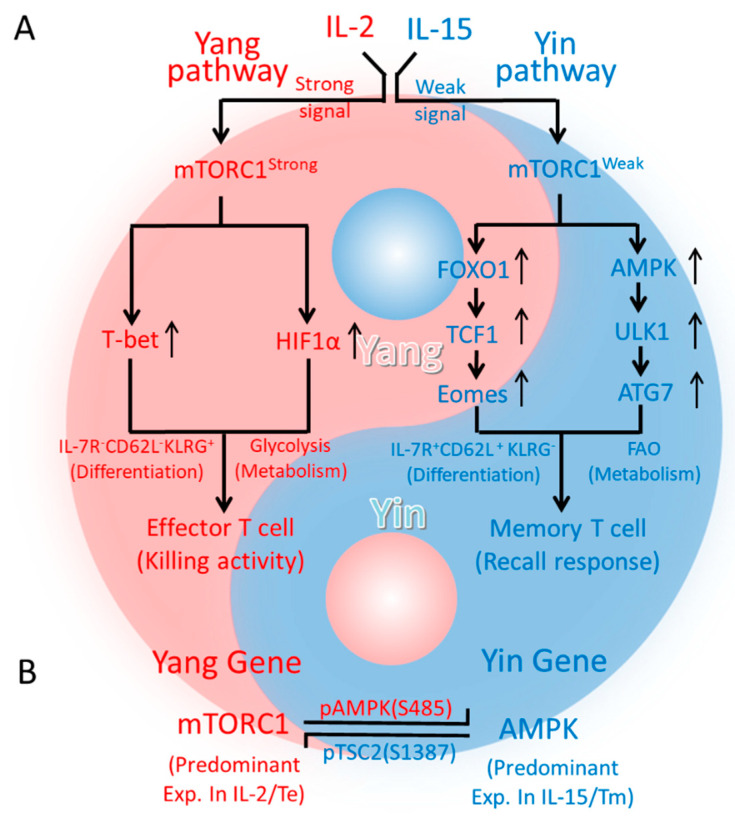
Schematic diagram of the interplay between Yin (AMPK1α) and Yang (mTORC1) energy sensors in T-cell differentiation. (**A**) IL-2 stimulates an mTORC1^Strong^ or Yang signal, leading to proliferative T_E_-cell formation via activation of the transcriptional T-bet (Yang) and the metabolic HIF-1α (Yang) pathways. In contrast, IL-15 induces an mTORC1^Weak^ or Yin signal leading to quiescent T_M_ cell formation via activation of the transcriptional FOXO1-TCF1-Eomes (Yin) and the metabolic AMPK-ULK1-ATG7 (Yin) pathways. (**B**) The graph shows the negative interplay between Yin (AMPK1α) and Yang (mTORC1) genes. In this figure, red represents Yang gene mTORC1 controlling T_E_-cell growth, differentiation and glycolytic fuel preference for energy production, while blue represents Yin gene AMPK1α regulating T_M_-cell quiescence and survival by using FAO for energy production.

In this study, we utilized a well-established protocol [16,18,19,20] for the in vitro preparation and characterization of IL-2/T_E_ and IL15/T_M_ cells as a working platform to explore molecular pathways central to IL-15-induced T-cell memory. The IL-2/T_E_ and IL-15/T_M_ cells were systematically characterized by Western blotting, flow cytometry, confocal and electron microscopy and Seahorse-assay analyses. Consistent with previous reports [12,21,38], we demonstrated that IL-2 and IL-15 trigger different strengths of mTORC1 (mTORC1^Strong^ and mTORC1^Weak^, respectively) signaling, which result from the persistent expression of IL-2Rα in IL-2/T_E_ cells and the transitional expression of IL-15Rα in IL-15/T_M_ cells. We further demonstrate that IL-15/T_M_ cells with mTORC1^Weak^ signaling up-regulate the transcription factors FOXO1, TCF1 and Eomes essential for T_M_-cell differentiation while down-regulating the abundance of the transcription factor T-bet required for T_E_-cell differentiation. IL-15/T_M_ cells with mTORC1^Weak^ signaling also up-regulate the energy sensor AMPKα1 and the AMPKα1-controlled markers of autophagy ULK1 and ATG7, mitochondrial fusion OPA1 and mitochondrial biogenesis TFAM, AQP9, CPT1α and Complex I. IL-15/T_M_ cells with mTORC1^Weak^ signaling also rely on FAO and down-regulate the expression of the transcription factor HIF-1α required for glycolysis. Consistent with these findings, we observed the reciprocal expression profile for transcription factors and their downstream targets in T_E_ cells with mTORC1^Strong^ signaling. In addition, IL-15/T_M_ cells exhibit long-term survival, whereas IL-2/T_E_ cells are short-lived post transfer into mice. Our data collectively indicate that IL-15/mTORC1^Weak^ signaling induces T_M_-cell formation in IL-15/T_M_ cells via activation of the transcriptional FOXO1-TCF1 Eomes and metabolic AMPK-ULK1-ATG7 pathways while IL-2/mTORC1^Strong^ signaling promotes T_E_-cell differentiation in IL-2/T_E_ cells via activation of the transcriptional T-bet and metabolic HIF-1α pathways (Figure 6A). These findings represent a novel molecular mechanism for T-cell memory in the LCD model [4], and are consistent with our recent reports showing that the pro-survival cytokine IL-7 or an inflammatory IL-2/rapamycin combinatorial treatment triggers mTORC1^Weak^ signaling to promote T-cell memory via the same transcriptional FOXO1 and metabolic AMPKα1 pathways [18,39].

To assess the importance of the metabolic AMPKα1 pathway in IL-15-induced T-cell memory, we repeated our comprehensive suite of analyses using IL-15/T_M_ cells lacking AMPKα1. Interestingly, AMPKα1 deficiency attenuates activation of the autophagic marker ULK1 and impairs mitochondrial biogenesis, leading to the up-regulation of mTORC1 and HIF-1α activity and a metabolic switch from FAO to glycolysis. The observed change in fuel preference in the absence of AMPKα1 may reflect the loss of its ability to inhibit mTORC1 via activation of the mTORC1 suppressor tuberous sclerosis-2 (TSC2) [40,41]. In addition, AMPKα1 deficiency also reduces T_M_-cell survival and recall responses in AMPKα1 KO IL-15/T_M_ cells, which is consistent with some previous reports [16,42]. Given that their elevated mitochondrial content confers T_M_ cells with a bioenergetic advantage that affords for robust recall responses upon antigen recounter [43], the reduced recall responses may be attributable to the lower mitochondrial mass of AMPKα1 KO IL-15/T_M_ cells compared to WT IL-15/T_M_ cells. Taken together, our data indicate that the metabolic AMPKα1-ULK1-ATG pathway is critical and indispensable for IL-15-induced T-cell memory.

The theory of Yin (negative regulation) and Yang (positive regulation) with a negative feedback interplay represents one of the most fundamental principles in traditional Chinese medicine. This theory has been applied to interpret distinct molecules (CTLA and CD28 or IFN-γ), cells (Treg and Th1) and metabolic fuel preferences (AMPKα1-mediate FAO and mTORC1-mediated glycolysis) as Yin and Yang, respectively, in the immune system, where Yin represents immune regulation/tolerance and cell quiescence, while Yang represents immune initiation/activation and cellular growth/function [44,45,46,47]. Based upon this principle, the pro-survival cytokines IL-7/IL-15 and IL-7/15-stimulated T_M_ cells and the pro-inflammatory cytokine IL-2 and IL-2-stimulated T_E_ cells would belong to Yin and Yang cytokines and T cells, respectively. However, how this theory guides immune responses at the molecular level is still unknown. In this study, our findings show how AMPKα1 acts as the Yin gene and mTORC1 acts as the Yang gene in regulating CD8^+^ T-cell differentiation into T_M_ and T_E_ cells, respectively. Specifically, IL-15-stimulation results in mTORC1^Weak^ or Yin signaling, leading to activation of the transcriptional FOXO1-TCF1-Eomes (Yin) and metabolic AMPKα1-ULK1-ATG7 (Yin) pathways and cell differentiation into IL-15/T_M_ cells that are quiescent and utilize the FAO metabolism for energy production (Figure 6A). In contrast, IL-2-stimulation results in mTORC1^Strong^ or Yang signaling, leading to activation of the transcriptional T-bet (Yang) and metabolic HIF-1α (Yang) pathways and cell differentiation into IL-2/T_E_ cells that are proliferative, effective at killing tumor or infected cells, and utilize glycolysis for energy production (Figure 6A).

The Yin and Yang theory argues for negative interplay between both elements. Indeed, it has been demonstrated that the Yang gene mTORC1 and Yin gene AMPKα1 do suppress one another, and that mTORC1 (Yang gene) and FOXO1 (Yin gene) also inhibit each other [48]. While there is, therefore, ample support for the principle that the AMPKα1/FOXO1 (Yin) and mTORC1 (Yang) genes interplay via a negative feedback loop, the underlying molecular mechanisms are unknown. In this study, we demonstrate that IL-15-stimulated mTORC1^Weak^ signaling induces IL-15/T_M_ cell formation by activating the transcriptional FOXO1 and metabolic AMPKα1 networks while concomitantly inhibiting the transcriptional T-bet and metabolic HIF-1α networks. In contrast, the IL-2-stimulated mTORC1^Strong^-signaling that induces IL-2/T_E_ cell formation relies on the reciprocal regulation of this transcriptional and metabolic circuitry. Our data show that Yang or mTORC1^Strong^ signaling inhibits the Yin gene AMPKα1 expression in Yang gene mTORC1 dominant IL-2/T_E_ cells, while mTORC1^Weak^-induced Yin gene AMPKα1 expression promotes T-cell memory in Yin gene AMPKα1 dominant IL-15/T_M_ cells, indicating the Yin gene AMPKα1 and Yang gene mTORC1 interplay via a negative feedback loop in CD8^+^ T-cell differentiation (Figure 6B). In addition, we show that the phosphorylation of AMPKα1 at S_485_, which depends on mTORC1 and blocks its activating phosphorylation at T_172_ [49], is enriched in mTORC1-dominant IL-2/T_E_ cells, and IL-15/T_M_ cells up-regulate the abundance of mTORC1 suppressor pTSC2 (S_1387_) downstream of AMPKα1 [40,41] to weaken mTORC1 signaling. Therefore, our data collectively imply that a negative feedback mechanism exists between the Yin gene AMPKα1 and Yang gene mTORC1 that is vital to CD8^+^ T cell memory (Figure 6B).

Taken together, we provide the first evidence that the pro-survival cytokine IL-15-stimulated mTORC1^Weak^ signaling controls CD8^+^ T-cell memory via activation of the transcriptional FOXO1-TCF1-Eomes and metabolic AMPKα1-ULK1-ATG7 pathways. This finding may represent a broader molecular mechanism employed by pro-survival cytokines that underlies the LCD model of T-cell memory formation and, as such, has the potential to greatly impact vaccine development and immunotherapy for cancer and infectious diseases.

## 4. Materials and Methods

### 4.1. Mice

The animal protocol (#20180065) used in this study was approved by the Animal Use and Care Committee at the University of Saskatchewan. Mice used in this study include B6.SJL-Ptprca Pepcb/BoyJ (B6.1, CD45.1^+^, #2014), C57BL/6 (B6, CD45.2^+^, #000664), ovalbumin (OVA)-specific T-cell receptor (TCR) transgenic OTI on a B6 background (CD45.2^+^ B6/OTI, #003831), *CD4*Cre (#022071) and *AMPKα1*^flox/flox^ (#014141) and were obtained from the Jackson Laboratory (Bar Harbor, MA, USA). All mice were maintained in the animal facility at the University of Saskatchewan. CD45.1^+^ B6.1 mice were intercrossed with B6/OTI (CD45.2^+^) mice to generate CD45.1^+^/45.2^+^ B6.1/OTI mice [18]. The OTI/*AMPKα1* KO mice were generated by cross breeding using *AMPKα1^flox/flox^*, *CD4*Cre and OTI mice as previously described [18].

### 4.2. Lymphocyte Preparation

Mouse splenocytes were prepared by repeated organ maceration with a 70 µm cell strainer into RPMI 1640 medium supplemented with 10% (*v*/*v*) fetal calf serum (FCS). Mouse peripheral blood samples were obtained by nicking the lateral tail vein and collecting blood into tubes containing heparin (BD Biosciences, San Jose, CA, USA). Red blood cells in splenocytes and peripheral blood were lysed for 5 min in Ack lysing buffer (150 mM NH_4_Cl, 10 mM KHCO_3_, and 0.1 mM EDTA) at room temperature. After quenching with RPMI 1640 medium and then a centrifugation step, cell pellets were re-suspended in 5 mL of RPMI 1640 medium for further purification of CD8^+^ T cells.

### 4.3. Cell Culture

CD8^+^ T cells were purified from mouse splenocytes using an Easysep CD8^+^ T-cell purification kit (StemCells Technologies, Vancouver, BC, Canada) according to the manufacturer’s protocol to yield T-cell populations comprising ~95% CD8^+^ T cells. CD8^+^ T cells purified from B6.1/OTI mouse splenocytes were cultured in RPMI 1640 medium containing 10% FCS, 100 U/mL IL-2 (Peprotech, Rocky Hill, NJ, USA), 2-mercaptoethanol (2-ME, 50 µM) and ovalbumin (OVA)_257-264_ peptide (OVA1, SIINFEKL; 0.1 nM) for 3 days. The activated T cells were then re-cultured in RPMI 1640 medium containing 10% FCS and IL-2 (100 U/mL) or IL-15 (10 ng/mL) for another 2 days to generate IL-2-stimulated effector T (IL-2/T_E_) or IL-15-stimulated memory T (IL-15/T_M_) cells [18] for adoptive transfer and various in vitro experiments including flow cytometry, Western blotting, confocal and electron microscopy and Seahorse-assay analyses.

### 4.4. Adoptive T Cell Transfer into B6 or B6.1 Mice Followed by Flow Cytometry Analyses for T Cell Survival

An equal number (5 × 10^6^ cells/mouse) of CD8^+^ IL-2/T_E_ or IL-15/T_M_ cells derived from CD45.1^+^/CD45.2^+^ B6.1/OT1 mice was intravenously (i.v.) injected into the tail vein of CD45.2^+^ B6 mice, and their survival was quantified kinetically by flow cytometry analysis of mouse peripheral blood samples. In another experiment, an equal number (5 × 10^6^ cells/mouse) of a 1:1 mixture of CD8^+^ IL-15/T_M_ and AMPKα1 KO IL-15/T_M_ cells derived from wild-type (WT) CD45.1^+^/CD45.2^+^ B6.1/OT1 and AMPKα1 KO CD45.2^+^ B6/OT1 mice, respectively, were followed post transfer by kinetic flow cytometry analyses using anti-CD45.1 and anti-CD45.2 antibody staining to independently measure the cell phenotype and survival of the two types of transferred T cells in the same mouse (see the section of flow cytometry). To investigate T_M_ cell recall responses, mice were boosted with an i.v. injection of 2000 colony-forming units (CFUs) of *Listeria monocytogenes* rLmOVA at day 30 post T cell transfer, followed by flow cytometry analysis using mouse peripheral blood samples at day 4 post rLmOVA boost [18].

### 4.5. Flow Cytometry

To quantify the expression of T-cell surface molecules, the in vitro prepared IL-2/T_E_ and IL-15/T_M_ cells or T cells derived from peripheral blood samples were stained with a mixture of some of the antibodies described below (each at 1:100) in 100 µL of flow wash buffer (FWB; 2% FCS and 0.1% sodium azide in PBS). After a 30 min incubation on ice in the dark, cells were washed twice with FWB and then analyzed by flow cytometry. The antibodies used for cell surface staining were purchased from BioLegend (San Diego, CA, USA): PE-Cy5-CD8 (clone53-6.7), FITC-CD45.1 (clone A20), Alexa Fluor 700-CD45.2 (clone 104), allophycocyanin-KLRG1 (clone 2F1), allophycocyanin-A750-CD62L (clone MEL-14), Brilliant Violet 510-IL-2Ra (clone A7R34) and PE-IL-15Ra (clone A7R34). For measurement of the mitochondrial mass, IL-2/T_E_ and IL-15/T_M_ cells were stained with 10 nM MitoTracker Green (Thermo Fisher Scientific, Waltham, MA, USA) for 15 min at 37 °C, washed three times with PBS and then analyzed by flow cytometry, as previously described [18]. Flow cytometry analyses were performed on a Cytoflex Multicolour Flow (Beckman, San Diego, CA, USA). Data were analyzed by FlowJo 10 (FlowJo, LLC, Ashland, OR, USA).

### 4.6. Confocal and Electron Microscopy Imaging

In vitro prepared IL-2/T_E_ and IL-15/T_M_ cells were added to an 8-well Lab-Tek II chamber slide (ThermoFisher Scientific, Waltham, MA, USA) coated with poly-D-lysine (Sigma, Oakville, ON, Canada), washed twice with PBS and incubated with a FITC-CD8 antibody (clone 53-6.7, BioLegend) at 4 °C for 30 min. T cells were then fixed and permeabilized in 1 mL fixation/permeabilization buffer (eBioscience) at 4 °C for 1 h, washed once with 1× permeabilization buffer, and incubated with primary antibodies against FOXO1 (clone 29H4) or TCF1 (clone 6309, Cell Signaling, Danvers, MA, USA) in 100 μL 1× permeabilization buffer [18] to visualize their intracellular localization. T cells were then stained with a PE-goat anti-rabbit IgG secondary antibody (BioLegend) for 30 min at room temperature and washed with PBS. After the chamber wall was removed, an antifade mountant with DAPI (ThermoFisher Scientific) was added and cover slips were applied [18]. MitoTracker and Hoechst staining were also conducted to visualize the mitochondrial content, according to the manufacturer’s instructions (Life Technologies, Oakville, ON, Canada). The images were taken with the ZEISS LSM confocal microscope (Carl Zeiss, Oberkochen, Germany). Confocal images were analyzed using ZEN imaging software [18]. For electron microscopic imaging of mitochondria, T cell pellets (2 × 10^6^ T cells/each) were fixed in 2% paraformaldehyde and 2.5% glutaraldehyde in 100 mM sodium cocodylate, washed in cocodylate buffer and fixed in 1% osmium tetroxide. After washing with water, samples were stained with 1% aqueous uranyl acetate for 1 h and washed again. After dehydration in ethanol and embedding in Eponate 12 resin (Ted Pella, Redding, CA, USA), samples were sectioned and imaged using a JOEL 1200 EX transmission electron microscope (JOEL Ltd., Tokyo, Japan) [18].

### 4.7. Western Blot Analysis

T cells were lysed in Ripa buffer containing a protease inhibitor and phosphatase inhibitor cocktail (Thermo Fisher Scientific, Waltham, MA, USA). Cell lysates were centrifuged at 4 °C at 12,000× *g* for 10 min, separated by SDS-PAGE and transferred onto a PVDF membrane. The membrane was blocked with 5% BSA in PBS containing 0.05% Tween-20 and incubated with various antibodies recognizing pAMPKα1 (T_172_), pAMPKα1 (S_485_), pS6 (S_235/236_), peIF4E (S_209_), pULK1 (S_555_), ATG7, AQP9, PGC1α, TFAM, OPA1, pDRP1 (S_616_), ID2, ID3, cMyb, cMyc, Blimp1, T-bet, ZEB2, FOXO1, TCF1, TRAF6, pTSC2 (S_1387_), Eomes, HIF-1α and β-actin (Cell Signaling Technology) and the Complex I subunit NDUFA9 (Abcam, Cambridge, MA, USA). The membranes were imaged using a BioRad Chemidoc MP (Bio-Rad, Hercules, CA, USA) after a second incubation step with a horseradish peroxidase-conjugated goat anti-rabbit IgG or goat anti-mouse IgG (Cell Signaling) secondary antibody [18].

### 4.8. NAD+/NADH Quantification

NAD+/NADH quantification colorimetric kits (BioVision, Milpitas, CA, USA) were used to measure NAD+ and NADH [18]. Briefly, 2 × 10^5^ IL-2/T_E_ and IL-15/T_M_ cells were pelleted and extracted with 400 μL of NADH/NAD extraction buffer by homogenization on ice. The extract was then vortexed and centrifuged at 14,000 rpm for 5 min. The supernatants were filtered through 10 kDa molecular weight cutoff filters (Millipore, Billerica, MA, USA). Then, 50 μL of supernatant from each sample was added to a 96-well plate to detect total NADt (NADH and NAD). For making a standard NADH curve, the extracted samples were heated to 60 °C for 30 min, and then transferred to 96-well plates to detect NADH [18]. The NAD/NADH ratio was calculated as (NADt-NADH)/NADH.

### 4.9. Seahorse-Assay Analysis

T cells were resuspended in serum-free Seahorse XF RPMI medium (Agilent, Lexington, MA, USA) and then plated into Seahorse XF cell culture microplates (1.5 × 10^5^ cells per well) coated with poly-D-lysine (Sigma-Aldrich, Oakville, ON, Canada) for T-cell attachment. A mitochondrial stress test was performed on a Seahorse XF analyzer (Agilent, Lexington, MA, USA) to measure OCR (pmol min^−1^) under basal conditions and upon sequential injection of oligomycin (1.5 μM), FCCP (2.5 μM), and rotenone/antimycin A (0.5 μM) (Agilent, Lexington, MA, USA) [18]. The following conditions were used in experiments with the Seahorse system: 3 min mixture; 0 min wait; and 3 min measurement.

### 4.10. Data Analyses

Data are expressed as the mean (with SD). Statistical analyses were performed using Prism 8 (GraphPad, La Jolla, CA, USA), and significant differences between groups in our study were detected with the Student *t* test. Probability values of *p* < 0.05, and *p* < 0.01 are considered statistically significant and very significant.

## Data Availability

Data are available on request from the corresponding author.

## Abbreviations

AMPKα1	adenosine monophosphate-activated protein kinase-α1
AQP9	aquaporin-9
ATG7	autophagy-related gene-7
CPT1α	carnitine palmitoyl transferase-1α
DRP1	dynamin-related protein-1
ECAR	extracellular acidification rate
ETC	electron transport chain
FAO	fatty acid oxidation
FOXO1	forkhead box-O-1
HIF-1α	hypoxia-inducible factor-1α
Id2/3	inhibitor of DNA binding-2/3
JAK3	Janus kinase-3
KLRG1	killer cell lectin-like receptor subfamily G member-1
KO	knockout
LAL	lysosomal acid lipase
LC3I/II	microtubule-associated protein light chain-3 I/II
LCD	linear cell differentiation
MPEC	memory precursor effector cell
mTORC1	mammalian target of rapamycin complex-1
OCR	O_2_ consumption rate
OPA1	optic atrophy-1
OXPHOS	oxidative phosphorylation
PGC1α	peroxisome proliferator-activated receptor-γ coactivator-1α
PI3K	phosphatidylinositol-3 kinase
γ_c_	common γ-chain
ROS	reactive oxygen species
SLEC	short-lived effector cell
SRC	spare respiratory capacity
TAG	triacylglycerol
TCA	tricarboxylic acid
TCF1	T cell factor-1
T_E_ cell	effector T cell
TFAM	mitochondrial transcription factor-A
T_M_ cell	memory T cell
TNF	tumor necrosis factor
TRAF6	TNF receptor-associated factor-6
ULK1	Unc-51-like autophagy-activating kinase-1
WT	wild type
